# Influence of the Ti(O*i*Pr)_4_: methacrylic acid ratio on the formed oxo/alkoxo clusters

**DOI:** 10.1007/s00706-015-1443-6

**Published:** 2015-04-02

**Authors:** Matthias Czakler, Christine Artner, Ulrich Schubert

**Affiliations:** Institute of Materials Chemistry, Vienna University of Technology, Vienna, Austria

**Keywords:** Titanium alkoxides, Methacrylate ligands, Structure analysis

## Abstract

**Abstract:**

When Ti(O*i*Pr)_4_ was reacted with increasing proportions of methacrylic acid (McOH), the compounds Ti_2_(O*i*Pr)_6_(OMc)_2_(*i*PrOH), Ti_6_O_4_(O*i*Pr)_8_(OMc)_8_, Ti_9_O_8_(O*i*Pr)_4_(OMc)_16_, and Ti_8_O_8_(OMc)_16_ were obtained in sequence. This allowed conclusions on the relative ratio of substitution and hydrolysis reactions, the latter being due to ester formation between the acid and cleaved alcohol. This ratio is also influenced by the reaction temperature, since Ti_4_O_2_(O*i*Pr)_6_(OMc)_6_ was formed instead of Ti_6_O_4_(O*i*Pr)_8_(OMc)_8_ at lower temperature with the same precursor ratio.

**Graphical abstract:**

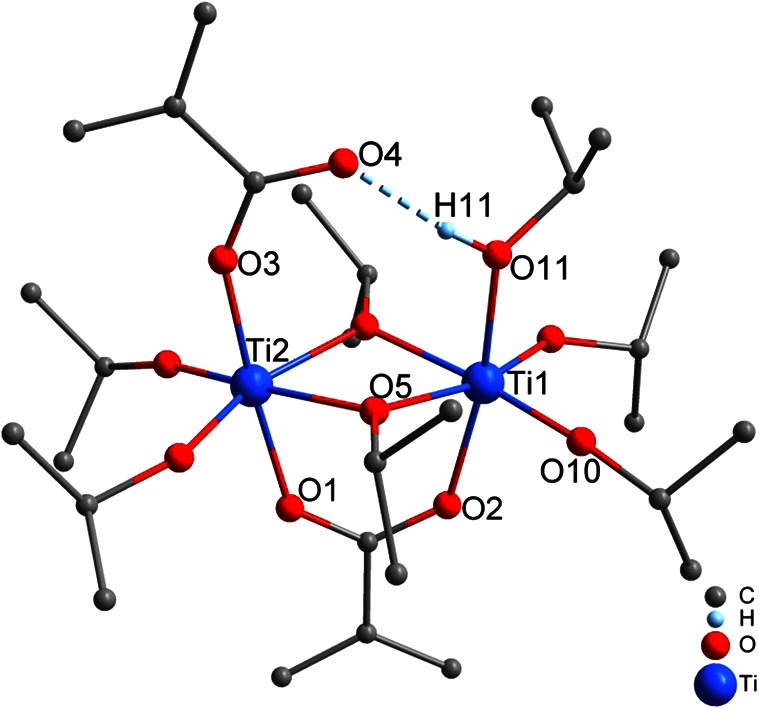

## Introduction

Metal oxo clusters, which are obtained by partial hydrolysis of a hydrolysable metal precursor, are valuable building blocks for inorganic–organic hybrid materials or metal–organic framework structures [1–4]. An efficient route is reaction of (functional or non-functional) carboxylic acids with early transition metal alkoxides. The carboxylic acids not only provide carboxylate ligands for stabilization of the metal-oxo cluster cores but also act as an in situ water source through esterification reactions.

A reasonable sequence of reactions is that (i) an OR group of the metal alkoxide, [M(OR)_x_]_n_, is substituted by a carboxylate ligand to give [M(OR)_x−1_(OOCR′)]_m_ and ROH; (ii) the eliminated alcohol reacts with carboxylic acid to give an ester and water; (iii) the thus generated water hydrolyses part of the remaining OR groups to give a cluster M_a_O_b_(OR)_c_(OOCR)_d_. Although the first step is necessary for the overall reaction, only few (non-hydrolyzed) carboxylate-substituted metal alkoxides are known. For titanium alkoxides, which are subject of this article, carboxylate-substituted metal alkoxide derivatives were only isolated from reactions with the bulky pivalic acid [5]. Another derivative was obtained by reaction with phthalic anhydride, where only substitution, but no hydrolysis is possible [6]. Ester formation (step ii) is easily monitored by IR spectroscopy. The isolation of a few polynuclear compounds with coordinated water [7, 8] is strong evidence that water is concomitantly formed (instead of oxo group formation by a non-hydrolytic process). Cluster formation (step iii) must be a stepwise process, but details, such as intermediates, are still unknown. Empirical evidence suggests, however, that competition between the two carboxylic acid-consuming reactions, namely substitution and ester/water formation, is decisive for the outcome of the reaction. Any parameter influencing the relative reaction rate of the two processes, such as kind of OR group, acidity and steric bulk of the carboxylic acid, or the metal alkoxide:carboxylic acid ratio, influences the composition of the eventually formed cluster M_a_O_b_(OR)_c_(OOCR)_d_. Correspondingly, the degree of substitution (d:a ratio) and the degree of condensation (b:a ratio) are the two main parameters from which conclusions can be drawn on the cluster-forming processes.

A series of clusters was previously obtained from Zr(OBu)_4_ and methacrylic acid (McOH) when the molar ratio was varied. With a low proportion of McOH, the cluster Zr_6_O_2_(OBu)_10_(OMc)_10_ was obtained, with both a low O:Zr (0.33) and OMc:Zr ratio (1.66) [9], both limited by the available amount of McOH. A high McOH proportion resulted in the cluster Zr_4_O_2_(OMc)_12_ [10, 11] with a high degree of substitution (OMc:Zr = 3.0). The highest degree of condensation (O:Zr = 0.66 in Zr_6_(OH)_4_O_4_(OMc)_12_ [11]) was obtained for an intermediate Zr(OBu)_4_:McOH ratio, where the degree of substitution in the cluster (2.0) was not too high and thus allowed a higher degree of condensation.

The situation for titanium should be different, because OMc:Ti ratios >2.0 are not possible for compounds with octahedrally coordinated titanium atoms having bidentate carboxylate ligands. Reactions of Ti(OR)_4_ with carboxylic acids are particularly well investigated, and a great number of carboxylate-substituted titanium oxo/alkoxo clusters, with different OR/carboxylate combinations, are known [12–14]. In this article, the influence of a single parameter on the composition of the formed cluster was systematically investigated, namely the metal alkoxide:carboxylic acid ratio. All other parameters, especially the kind of metal alkoxide [Ti(O*i*Pr)_4_] and the kind of acid (methacrylic acid) were unvaried.

Methacrylic acid is frequently used because of its small, rigid shape and the polymerizable group (for the preparation of class II hybrid materials). The only structures reported until present, obtained from reaction of Ti(O*i*Pr)_4_ with methacrylic acid at different molar ratios, are Ti_4_O_2_(O*i*Pr)(OMc)_6_ (Ti(O*i*Pr)_4_:McOH ratio 1:2.3) [15] and Ti_8_O_8_(OMc)_16_ (ratio 1:4) [16] (Eq. (1)).

1 $$ {\text{Ti}}\left( {{\text{O}}i{ \Pr }} \right)_{ 4} + {\text{ CH}}_{ 2} = {\text{CMe}} - {\text{COOH}} \to {\text{Ti}}_{\text{a}} {\text{O}}_{\text{b}} \left( {{\text{O}}i{ \Pr }} \right)_{\text{c}} \left( {\text{OMc}} \right)_{\text{d}} + {\text{ McO}}i{ \Pr } $$

## Results and discussion

**Fig. 1:**
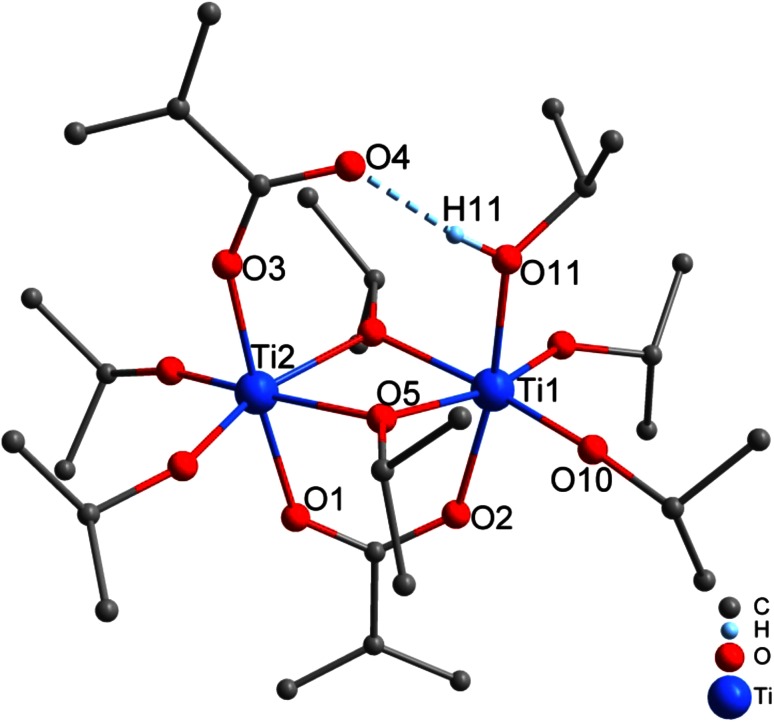
Molecular structure of Ti_2_(µ_2_-O*i*Pr)_2_(O*i*Pr)_4_(µ_2_-OMc)(OMc)(*i*PrOH) (**1**). Hydrogen atoms except that of the hydrogen bond (H11) were omitted for clarity

The dimeric complex Ti_2_(µ_2_-O*i*Pr)_2_(O*i*Pr)_4_(µ_2_-OMc)(OMc)(*i*PrOH) (**1**, Fig. 1) crystallized from an equimolar mixture of Ti(O*i*Pr)_4_ and McOH in 2-propanol at −10 °C. No esterification occurred at this temperature.

Compound **1** is isostructural to the other binuclear carboxylate-substituted titanium alkoxides mentioned above [5, 6]. The two octahedrally coordinated titanium atoms in **1** are bridged by two O*i*Pr and one OMc ligands. A second OMc ligand is η^1^-coordinated to one titanium atom and stabilized by a hydrogen bond to an isopropanol molecule coordinated to the other titanium atom. We have pointed out earlier that the combination of a η^1^ carboxylate ligand and a neutral proton-donating ligand connected through a hydrogen bond is structurally equivalent to a mono-anionic bidentate ligand [7, 17].

In the reaction leading to **1**, the carboxylic acid was completely consumed for substitution of O*i*Pr groups, and consequently no ester and cluster formation was possible. The crystals of **1** dissolved when the reaction solution was warmed to room temperature, and after 48 h at room temperature, **1** did not crystallize again when cooled to −10 °C. This indicates that the substitution reaction might be reversible, with subsequent initiating of cluster-forming reactions.

**Fig. 2:**
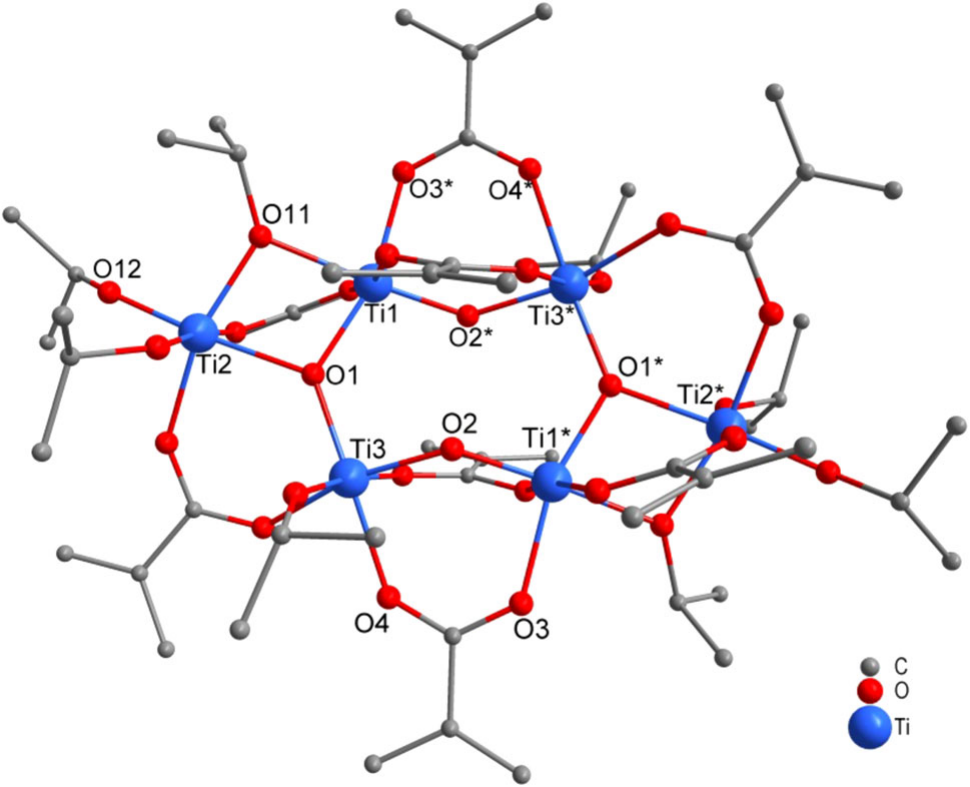
Molecular structure of Ti_6_(µ_3_-O)_2_(µ_2_-O)_2_(µ_2_-O*i*Pr)_2_(O*i*Pr)_6_(µ_2_-OMc)_8_ (**2**). Hydrogen atoms were omitted for clarity

When Ti(O*i*Pr)_4_ was reacted with two equivalents of McOH at room temperature, i.e., when the McOH proportion was increased, the centrosymmetric cluster Ti_6_(µ_3_-O)_2_(µ_2_-O)_2_(µ_2_-O*i*Pr)_2_(O*i*Pr)_6_(OMc)_8_ was obtained (**2**, Fig. 2). The two equivalents of McOH are consumed to two-thirds for substitution (O:Ti ratio 0.67) and to one-third for ester/water formation (OMc:Ti ratio 1.33). Isostructural clusters were previously obtained with other combinations of alkoxo and/or carboxylate ligands (see review articles [12–14]). Cluster **2** consists of six nearly coplanar titanium atoms, where two Ti_3_O units are connected through four OMc and two µ_2_-oxo bridges in total.

**Fig. 3:**
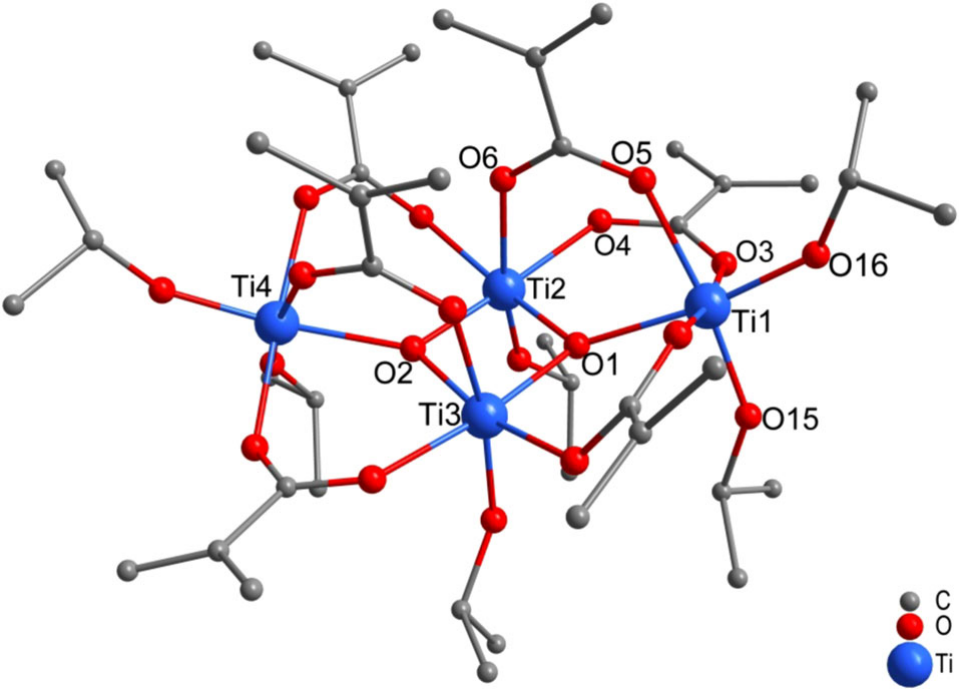
Molecular structure of Ti_4_O_2_(O*i*Pr)_6_(OMc)_6_ (**3**) [15]. Hydrogen atoms were omitted for clarity

A different cluster, viz. Ti_4_O_2_(O*i*Pr)_6_(OMc)_6_ (**3**, Fig. 3), was previously obtained for a very similar Ti(O*i*Pr)_4_:McOH ratio (1:2.3) [15]. Therefore, reaction of Ti(O*i*Pr)_4_ with two molar equivalents of McOH was repeated, but the mixture was stored at 4 °C. Because no crystals were obtained after 6 months, the mixture was warmed to room temperature; crystals of **3** were then formed after one additional month. The structure of **3** is simpler than that of **2**, as both oxygen atoms of the Ti_4_O_2_ core are µ_3_, all OMc ligands are bridging and all O*i*Pr ligands are terminal.

In both reactions, the one leading to **2** and the one leading to **3**, the two equivalents of McOH in the starting mixture were completely consumed. In **3**, however, 75 % were consumed for substitution and only 25 % for ester formation (O:Ti ratio of 0.5 and OMc:Ti ratio of 1.5), compared to 67:33 % in **2**. This shows that the reaction rate of ester formation was more strongly decreased by the lower temperature than that of the substitution reaction(s).

**Fig. 4:**
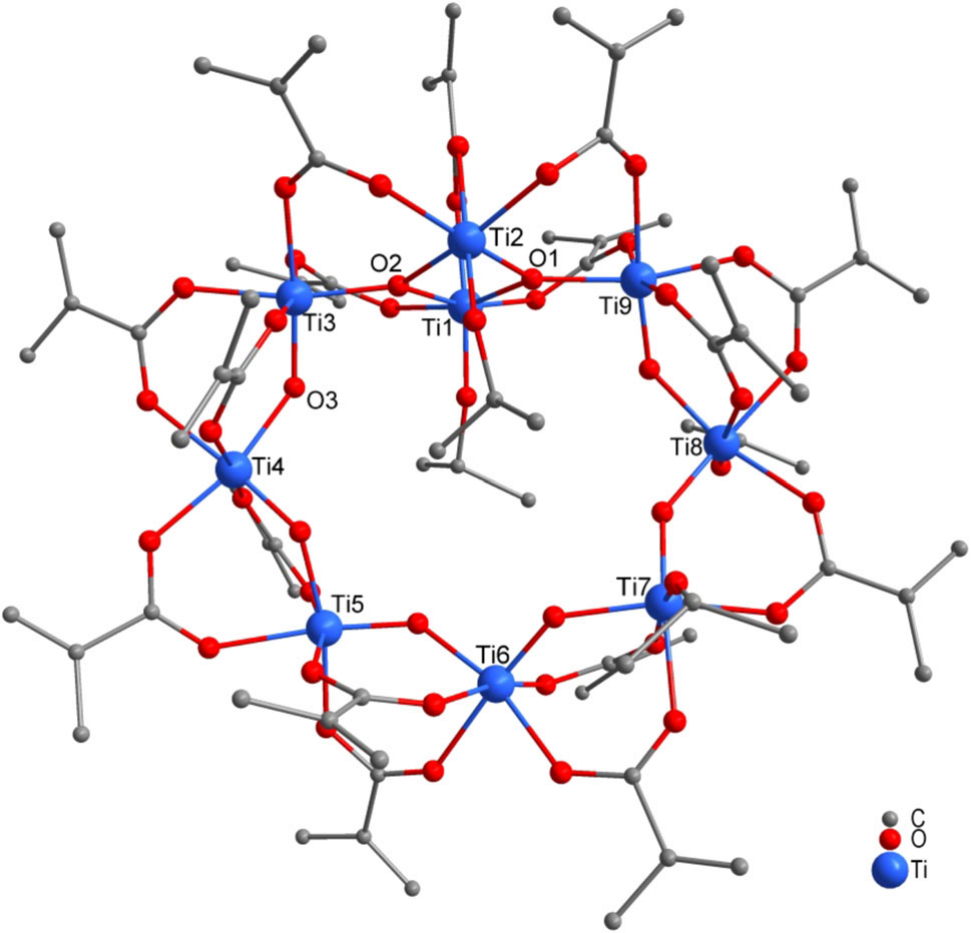
Molecular structure of Ti_9_(µ_3_-O)_2_(µ_2_-O)_6_(O*i*Pr)_4_(µ_2_-OMc)_16_ (**4**). Hydrogen atoms and solvent molecules were omitted for clarity

Reaction of Ti(O*i*Pr)_4_ with McOH in a ratio of 1:4 or 1:6 led to Ti_9_(µ_3_-O)_2_(µ_2_-O)_6_(O*i*Pr)_4_(µ_2_-OMc)_16_ (**4**, Fig. 4). While the carboxylic acid was completely consumed (for both substitution and ester formation) for Ti(O*i*Pr)_4_:McOH ratios of 1:1 and 1:2 (leading to compounds **1**–**3**), this is not possible for compound **4**, and part of McOH must remain unreacted. The higher proportion of McOH, however, results in a lower proportion of residual O*i*Pr groups.

An isostructural compound was previously obtained from the reaction of Ti(O*n*Pr)_4_ and McOH in a 1:4 ratio [18]. The structure of **4** is related to that of the known “titanyl carboxylates” Ti_8_(µ_2_-O)_8_(OOCR)_16_ (see below). Instead of a symmetric octanuclear ring system in the latter compounds, the structure of **4** has a “knot” as four of the nine [TiO_6_] octahedra (Ti1, Ti2, Ti3, and Ti9) are linked by two µ_3_-oxo bridges. All but two pairs of neighboring Ti octahedra are bridged by two methacrylate ligands. Ti7/Ti8 and Ti1/Ti2 are connected by only one methacrylate bridge; the second bridge is replaced by two O*i*Pr ligands (one at each Ti atom). This unusual ring structure was previously discussed in detail [18].

**Fig. 5:**
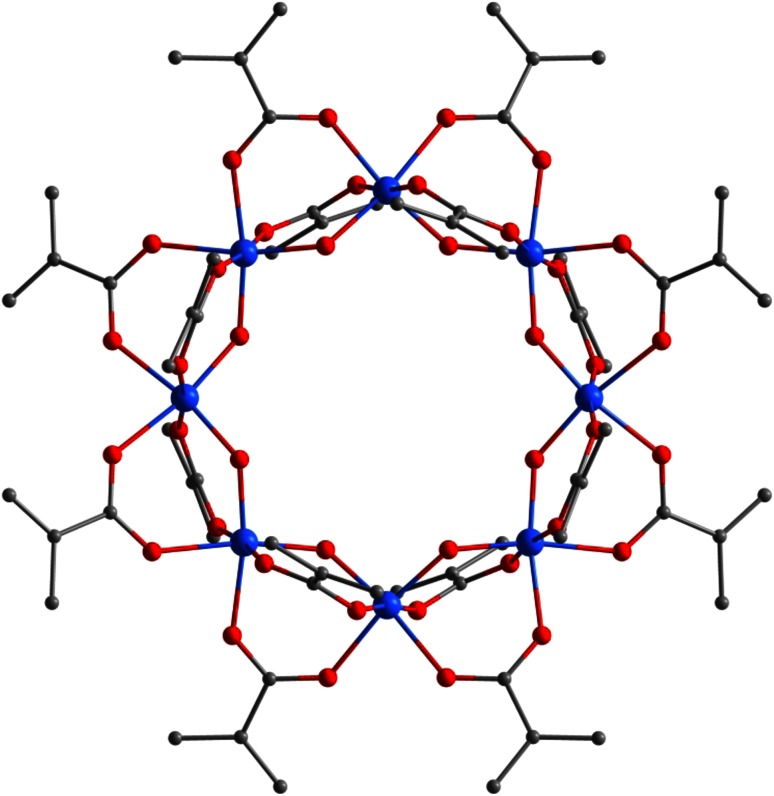
Molecular structure of Ti_8_(µ_2_-O)_8_(µ_2_-OMc)_16_ (**5**). Hydrogen atoms and solvent molecules were omitted for clarity

Further increase of the McOH proportion in the reaction mixture (ratio 1:8 or 1:16) led to the formation of “titanyl methacrylate”, Ti_8_(µ_2_-O)_8_(µ_2_-OMc)_16_ (**5**, Fig. 5), where all OR groups of the starting metal alkoxide were either substituted or hydrolyzed. The well-known Ti_8_O_8_ ring structure [16, 19–22] with *C*_4v_ symmetry is built from eight [Ti(µ_2_-O)(µ_2_-OMc)_2_] units which are mutually interconnected by one µ_2_-oxo and two OMc bridges each. The molecular structure of **5** is the same as that of another (orthorhombic) polymorph described earlier [16].

## Conclusions

When early transition metal alkoxides are reacted with carboxylic acid two carboxylic acid-consuming reactions compete with each other, namely substitution of part of the OR groups by carboxylate groups and ester formation with the eliminated alcohol. The thus generated water serves for (partial) hydrolysis of the OR groups.

Substitution without cocomitant hydrolysis was only achieved when Ti(O*i*Pr)_4_ was reacted with methacrylic acid in a 1:1 molar ratio at low temperature, where the rate of substitution is apparently much faster than that of ester + water formation (it is assumed that hydrolysis and condensation are fast reactions). Ti_2_(O*i*Pr)_6_(OMc)_2_(*i*PrOH) (**1**) appears to be kinetically favored, since it disappears when the reaction solution is warmed to room temperature.

Increasing the McOH:Ti(O*i*Pr)_4_ ratio at constant reaction conditions (room temperature) results in the formation of the oxo clusters Ti_6_O_4_(O*i*Pr)_8_(OMc)_8_ (**2**), Ti_9_O_8_(O*i*Pr)_4_(OMc)_16_ (**4**), and Ti_8_O_8_(OMc)_16_ (**5**) in sequence, where the number of residual OR per Ti is steadily decreased (Table 1). Analysis of the degree of condensation (O/Ti ratio) and degree of substitution (OMc/Ti ratio) of these clusters shows, however, that the latter is always two-times higher than the former, i.e., two-thirds of the reacted McOH are consumed for substitution and to one-third for ester/water formation.

**Table 1:** Comparison of the clusters **1**–**5**

McOH:Ti(O*i*Pr)_4_ ratio	Obtained cluster	Degree of condensation (O/Ti ratio)	Degree of substitution (OMc/Ti ratio)	Residual OR per Ti
1:1	Ti_2_(O*i*Pr)_6_(OMc)_2_(*i*PrOH) (**1**)	0	1	3
2:1	Ti_6_O_4_(O*i*Pr)_8_(OMc)_8_ (**2**)	0.67	1.33	1.33
2:1	Ti_4_O_2_(O*i*Pr)_6_(OMc)_6_ (**3**)	0.5	1.5	1.5
4:1 or 6:1	Ti_9_O_8_(O*i*Pr)_4_(OMc)_16_ (**4**)	0.89	1.78	0.44
8:1	Ti_8_O_8_(OMc)_16_ (**5**)	1	2	0

Changing the reaction temperature, however, also changes the relative rate of substitution and water formation (leading to condensation). With a constant McOH:Ti(O*i*Pr)_4_ ratio of 2, Ti_4_O_2_(O*i*Pr)_6_(OMc)_6_ (**3**) was formed at lower temperature instead of **2**. As already stated for **1**, the relative rate of the ester formation reaction is apparently slower when the temperature is decreased. This leads to a cluster (**3**) in which the degree of substitution is higher and the degree of condensation lower compared to **2**, which was formed at higher temperature.

## Experimental

All operations were carried out in a moisture- and oxygen-free argon atmosphere using standard Schlenk techniques. Ti(O*i*Pr)_4_ and methacrylic acid were obtained from ABCR and used as received. Isopropanol was dried by distillation from sodium metal twice. The solvents for NMR spectroscopy (Eurisotop) were degassed prior to use and stored over molecular sieve. ^1^H and ^13^C solution NMR spectra were recorded on a Bruker AVANCE 250 (250.13 MHz for ^1^H, 62.86 MHz for ^13^C) equipped with a 5 mm inverse-broadband probe head and a z-gradient unit.

### *Methacrylate*-*substituted titanium propoxide Ti*_*2*_*(OiPr)*_*6*_*(OMc)*_*2*_*(iPrOH) (****1****)*

Ti(O*i*Pr)_4_ (1 cm^3^, 3.45 mmol) was added to a solution of 291 mm^3^ methacrylic acid (3.45 mmol) in 3 cm^3^ of water-free isopropanol at 0 °C. A white precipitate formed immediately, which was dissolved by adding 1 cm^3^ of CH_2_Cl_2_. Crystals of **1** were obtained from the clear solution at −10 °C after 2 days. Yield 500 mg (43 %); ^1^H NMR (C_6_D_6_, 250 MHz): *δ* = 0.94–1.82 (m, 42H, CHC*H*_3_), 1.95–2.29 (m, 6H, CCH_3_), 4.51 (br, 2H, OCH), 4.88–5.33 (m, 5H, OCH), 5.42 (br, 2H, C=CH_2_), 6.47 ppm (br, 2H, C=CH_2_) ppm; ^13^C NMR (C_6_D_6_, 62.90 MHz): *δ* = 18.22 (br, C*C*H_3_), 24.98 (CH*C*H_3_), 25.37 (br, CH*C*H_3_), 77.90 (OCH), 72.3–79.36 (br, OCH), 125.1–126.8 (br, C*C*H_2_) ppm.

### *Methacrylate*-*substituted titanium oxo clusters Ti*_*6*_*O*_*4*_*(OiPr)*_*8*_*(OMc)*_*8*_*(****2****), Ti*_*9*_*O*_*8*_*(OiPr)*_*4*_*(OMc)*_*16*_*(****4****), and Ti*_*8*_*O*_*8*_*(OMc)*_*16*_*(****5****)*

Methacrylic acid (**2**: 582 mm^3^, 6.90 mmol; **4**: 1.75 cm^3^, 20.69 mmol; **5**: 2.33 cm^3^, 27.58 mmol) was added to 1 cm^3^ of Ti(O*i*Pr)_4_ (3.45 mmol) and the solution was stirred for 5 min. Crystals were obtained after 2–6 months at room temperature.

**2**: Yield 350 mg (40 %); ^1^H NMR (CDCl_3_, 200 MHz): *δ* = 1.00–1.44 (m, 48H, CHC*H*_3_), 1.68–1.96 (m, 24H, CCH_3_), 3.97 (m, *J* = 6.16 Hz, 1H, CH), 4.30 (m, *J* = 6.06 Hz, 1H, CH), 4.58–4.85 (m, 4H, CH), 4.93–5.12 (m, 2H, CH), 5.24–5.56 (m, 8H, CH_2_), 5.90–6.27 ppm (m, 8H, CH_2_) ppm; ^13^C NMR (CDCl_3_, 50.3 MHz): *δ* = 18.17, 18.26, 18.48, 18.58, 18.64, 18.74, 18.81 (C*C*H_3_), 24.41, 24.52, 24.90, 25.25, 25.33, 25.40, 25.47, 77.20, 78.13, 78.40, 78.85, 80.27, 81.62, 81.75 (CH*C*H_3_), 125.81, 126.96, 127.90 (CH_2_), 137.45, 137.61, 138.12, 138.55, 138.75, 139.19, 139.29, 139.40 (C=CH_2_), 172.77, 173.14, 174.31, 174.71, 175.12, 175.81 (COO) ppm.

**4**: Yield 330 mg (39 %); ^1^H NMR (CDCl_3_, 250 MHz): *δ* = 0.88–1.11 (m, 3H, CHC*H*_3_), 1.14 (d, *J* = 6.12 Hz, 6H, CHC*H*_3_), 1.17–1.60 (m, 15H, CHC*H*_3_), 1.64–1.96 (m, 48H, CCH_3_), 3.97 (m, *J* = 6.11 Hz, 1H, CH), 4.93–5.04 (m, 1H, CH), 5.20–5.58 (m, 16H, CH_2_), 5.61 (m, 3H, CH), 5.95–6.31 ppm (m, 16H, CH_2_) ppm.

**5**: Yield 80 mg (12 %); ^1^H NMR (CDCl_3_, 250 MHz): *δ* = 1.85–1.93 (m, 48H, CH_3_), 5.29–5.69 (m, 16H, CH_2_), 6.17–6.22 ppm (m, 16H, CH_2_) ppm; ^13^C NMR (CDCl_3_, 62.90 MHz): *δ* = 17.9–18.4 (CH_3_), 126.0–128.3 (CH_2_), 136.2–138.4 (C), 176.7 ppm (COO).

### *Methacrylate*-*substituted Ti*_*4*_*cluster Ti*_*4*_*O*_*2*_*(OiPr)*_*6*_*(OMc)*_*6*_*(****3****)*

Methacrylic acid (580 mm^3^, 6.87 mmol) was added to 1 cm^3^ of Ti(O*i*Pr)_4_ (3.45 mmol) at room temperature. After 5 min of stirring, the reaction vessel was stored at 4 °C. After 6 months, no crystals were obtained; therefore, the mixture was warmed to room temperature. After one additional month, crystals of **3** were obtained. Yield 200 mg (21 %). Spectroscopic data were already reported in Ref. [15].

### X-ray structure analyses

All measurements were performed at 100 K using MoK_α_ (*λ* = 71.073 pm) radiation. Data were collected on a Bruker Axs Smart Apex II four-circle diffractometer with κ-geometry, with φ and ω scans and different frame widths. The data were corrected for polarization and Lorentz effects, and an empirical absorption correction (Sadabs) was employed. The cell dimensions were refined with all unique reflections (Table 2). Saint Plus software (Bruker Analytical X-ray Instruments, 2007) was used to integrate the frames.

**Table 2:** Crystal data and structure refinement details for **1**, **2**, **4**, and **5**

	**1**	**2**	**4**	**5**
Emp. Formula	C_29_H_60_O_11_Ti_2_	C_56_H_96_O_28_Ti_6_	C_85.50_H_123_O_48.50_Ti_9_	C_64_H_80_O_40_Ti_8_
*M*_r_	680.57	1504.73	2357.94	1872.48
Crystal system	Monoclinic	Monoclinic	Triclinic	Monoclinic
Space group	*P*2_1_/*n*	*P*2_1_/*c*	*P* $\overline{1}$	*C*2/c
*a*/pm	1065.27(5)	1155.03(4)	1182.0(3)	2401.7(2)
*b*/pm	1819.00(8)	1895.81(5)	1626.7(3)	1662.8(2)
*c*/pm	1961.76(8)	1702.46(5)	3060.3(7)	2430.8(2)
*α*/deg	90	90	87.652(5)	90
*β*/deg	100.688(2)	105.181(1)	88.160(6)	90.459(6)
*γ*/deg	90	90	72.026(5)	90
*V*/pm^3^ × 10^6^	3735.4(3)	3597.8(2)	5591(2)	9707(1)
*Z*	4	2	2	4
*D*_x_/g cm^−3^	1.21	1.39	1.40	1.28
*µ*/mm^−1^	0.476	0.710	0.693	0.699
Crystal size/mm	0.5 × 0.5 × 0.5	0.4 × 0.25 × 0.2	0.2 × 0.15 × 0.05	0.65 × 0.64 × 0.33
No. msd. refl.	244,599	103,239	108,932	9827
Obs. refl. [*I* > 2*σ*(*I*)]	20,685	10,638	6190	7847
*θ*_max_/deg	44.89	33.50	20.02	26.38
*R* [*F* ^2^ > 2*σ*(*F*)], w*R* (*F* ^2^), *S*	0.0540, 0.0960, 1.108	0. 0361, 0. 0605, 1.032	0.0824, 0.1509, 1.087	0.1195, 0.2594, 3.502
Weighting scheme^a^	*w* = 1/[*σ* ^2^(*F*_0_^2^) + (0.0447*P*)^2^ + 1.7418*P*]	*w* = 1/[*σ* ^2^(*F*_0_^2^) + (0.0459*P*)^2^ + 1.6781*P*]	*w* = 1/[*σ* ^2^(*F*_0_^2^) + (0.1135*P*)^2^ + 28.5259*P*]	*w* = 1/[*σ* ^2^(*F*_0_^2^) + (0.0275*P*)^2^ + 11.5003*P*]
δ*ρ*_max_,_min_/e·10^−6^ pm^−3^	1.08, −0.52	0.96, −0.59	0.87, −0.91	1.45, −1.00

^a^ *P* = (*F*_0_^2^ + 2*F*_c_^2^)/3

The structures were solved by the Patterson method (Shelxs97 [23]). Refinement was performed by the full-matrix least-squares method based on *F* ^2^ (Shelxl97) with anisotropic thermal parameters for all non-hydrogen atoms. Hydrogen atoms were inserted in calculated positions and refined riding with the corresponding atom. Three O*i*Pr groups in **1** are disordered and were refined with around 80 % occupancy of the carbon atoms marked as “A” (e.g. C12A). One molecule of 2-propanol and two of methacrylic acid were found in the asymmetric unit of **4**, of which the 2-propanol was refined with 50 % occupancy. The electron density in the void opened by the ring of **5** could not be refined and was removed using the Squeeze function in Platon.

CCDC-1024252 (**1**), -1024253 (**2**), -1024254 (**4**), and -1024255 (**5**) contain the supplementary crystallographic data for this paper. These data can be obtained free of charge from The Cambridge Crystallographic Data Center via www.ccdc.cam.ac.uk/data_request/cif.
